# Transcriptome-Inferred metabolic subtypes define prognostic and immune ecosystems in osteosarcoma at single-cell resolution

**DOI:** 10.3389/fped.2026.1831516

**Published:** 2026-06-18

**Authors:** Li Hu, Dingsheng Zhang, Boyang Wang, Qian Liu, Xingyu Liao, Feiyang Qi, Linxi Chen, Huimin Liu, Zhiqing Zhao, Haijie Liang, Xingyu Liu, Zhiye Du, Rui Yang, Yi Yang, Yang Wang, Jichuan Wang

**Affiliations:** 1 Familial & Hereditary Cancer Center, Peking University Cancer Hospital & Institute, Key Laboratory of Carcinogenesis and Translational Research (Ministry of Education), Beijing, China; 2 Department of Medical Affairs, Peking University People’s Hospital, Beijing, China; 3 Musculoskeletal Tumor Center, Beijing Key Laboratory for Musculoskeletal Tumors, Peking University People’s Hospital, Beijing, China; 4 Multidisciplinary Diagnosis and Treatment Center for Bone Tumors, Peking University Shougang Hospital, Beijing, China; 5 Department of Orthopedics, Peking University First Hospital, Beijing, China; 6 Department of Orthopedic Surgery, UTHealth McGovern Medical School, University of Texas Health Science Center, Houston, TX, United States; 7 Comprehensive Clinical Trial Ward, Key Laboratory of Carcinogenesis and Translational Research (Ministry of Education), Peking University Cancer Hospital & Institute, Beijing, China

**Keywords:** cholesterol metabolism, consensus clustering, metabolic subtyping, osteosarcoma, oxidative phosphorylation, redox homeostasis, single-cell RNA sequencing, tumor microenvironment

## Abstract

Osteosarcoma exhibits substantial metabolic heterogeneity, yet existing classification schemes typically rely on narrow pathway windows and lack cellular-source attribution. Here, we integrated bulk transcriptomes from 157 osteosarcoma tumors (PKUPH-OS, *n* = 70; TARGET-OS, *n* = 87), quantified pathway-level activity via single-sample gene set enrichment analysis (ssGSEA) across curated metabolic gene sets, and performed consensus clustering to identify three stable metabolic subtypes: Cholesterogenic (C1), characterized by mTORC1-associated lipid biosynthesis and invasion-related signaling; Redox-Catabolic (C2), dominated by detoxification, glutathione/ROS buffering, and enhanced catabolic programs alongside immune engagement; and OXPHOS-Active (C3), featuring coupled mitochondrial oxidative phosphorylation and proliferative programs. Clinically, C2 displayed favorable survival (3-year overall survival: 90.8%), whereas C1 (60.0%) and C3 (61.9%) followed adverse trajectories (overall survival, *P* = 0.0037; progression-free survival, *P* = 0.011). Single-cell RNA sequencing of 16,276 cells with inferCNV-supported malignant-cell anchoring revealed distinct cellular origins: C1 signatures mapped predominantly to stromal/mesenchymal populations, C2 signatures to immune cells, and C3 signatures to malignant tumor cells. These observations apply within osteosarcoma the well-recognized principle that bulk transcriptomic signatures reflect cellular composition, providing an explicit cell-of-origin map of metabolic subtypes that supports niche-resolved risk stratification and cell-aware therapeutic hypothesis generation.

## 1 Introduction

Osteosarcoma is a biologically aggressive malignancy (1, 2) in which clinical trajectories often diverge despite apparently similar baseline presentation. Conventional clinicopathologic variables can provide coarse risk estimates (3, 4), yet they frequently fall short when asked to explain why some tumors relapse early or progress under otherwise standard treatment pressures. In practice, the most consequential heterogeneity is often functional: how tumor tissue allocates resources for growth, tolerates oxidative and xenobiotic stress, and adapts to fluctuating nutrient and oxygen availability. A stratification axis that is close to phenotype—mechanistically interpretable and measurable from routine molecular profiles—would therefore be valuable for clarifying risk and generating testable therapeutic hypotheses.

Metabolic organization offers a plausible route to such functional stratification. Among the omics layers, metabolism is often viewed as especially proximal to phenotype (5–7) because it reflects the integrated output of genetic and epigenetic programs, microenvironmental constraints, and cellular state transitions. Importantly, cancer metabolism is rarely a single-pathway toggle (8–10); instead, tumors tend to reassemble multiple modules—mitochondrial energy production, lipid handling, redox buffering, and anabolic precursor supply—into coordinated states that support specific survival strategies. In osteosarcoma, an expanding literature has begun to formalize this idea using metabolism-related features to define groups or risk scores, often accompanied by immune-context annotation. Examples include metabolism-related signatures linked to prognosis and immune microenvironmental estimates (11, 12), glycolysis–immune frameworks (13, 14) that associate glycolytic status with survival and immune infiltration patterns, and pathway-focused designs using glycolysis and cholesterol synthesis to stratify clinical outcome (15) while profiling immune checkpoint and antigen-presentation signals. Parallel efforts have emphasized lipid-metabolism signatures (16) and their coupling to immune states, as well as mitochondrial/OXPHOS-associated programs (17) that appear to partition osteosarcoma into biologically and clinically distinct categories. Together, these studies support the notion that metabolic heterogeneity in osteosarcoma is clinically relevant and frequently entangled with the tumor immune microenvironment.

However, current work still leaves two practical problems unresolved. First, many studies operationalize “metabolic subtyping” through a relatively narrow window—glycolysis, lipid metabolism, lactate/fatty-acid modules, copper-related metabolism, or selected mitochondrial gene sets—such that the resulting group labels are strongly conditioned on the predefined feature space and the particular scoring or clustering scheme. This pathway-anchored fragmentation makes it difficult to reconcile subtype definitions across studies and, more importantly, risks missing alternative metabolic configurations that may coexist within osteosarcoma tissue. Broader, pathway-level architectures—designed to survey metabolism as an interacting system rather than a single axis—have been proposed as a way to place these observations into a more coherent landscape. Second, and more limiting for biological interpretation, tissue-level metabolic signals—whether measured directly by conventional metabolomics or inferred from bulk molecular profiles—often lack clear cellular provenance. Tumor specimens contain malignant cells interwoven with immune and stromal compartments, each carrying distinct baseline metabolic programs; thus, a tissue-level “metabolic subtype” may reflect tumor-intrinsic rewiring, shifts in microenvironmental composition, or both. Without cell-resolved attribution, it becomes difficult to translate subtype labels into mechanism or intervention logic, because a program driven by infiltrating myeloid cells is fundamentally different from the same program executed by malignant cells. Although integrated bulk–single-cell analyses have been attempted (11, 18), they often begin from a limited set of metabolic themes, leaving the cellular origin of tissue-level metabolic heterogeneity incompletely clarified in osteosarcoma.

Motivated by these gaps, we designed a pathway-level and cell-aware framework to delineate osteosarcoma metabolic states and connect them to clinical phenotype and microenvironmental context. We integrated an institutional bulk transcriptomic cohort (PKUPH-OS) with a public bulk dataset (TARGET-OS), yielding a total of 157 tumors, and quantified sample-level activity profiles across curated metabolic pathways using ssGSEA before unsupervised consensus clustering for subtype discovery. Downstream analyses were structured to preserve interpretability across modules, including transcriptional program annotation and complementary tumor microenvironment inference. Critically, we leveraged a single-cell atlas with inferCNV-supported malignant-cell anchoring to project bulk-derived subtype signatures onto resolved cell populations, enabling direct tracing of the dominant cellular sources of each metabolic state rather than assuming tumor-intrinsic origin. This integrated design aims to move osteosarcoma metabolic classification toward a system-level taxonomy with explicit cellular provenance, thereby strengthening biological interpretability and sharpening the space of plausible therapeutic vulnerabilities.

## 2 Materials and methods

### 2.1 Study cohorts

Our retrospective cohort comprised 70 osteosarcoma patients treated at Peking University People's Hospital from June 2012 to December 2023, with adequate tumor tissue for RNA extraction and complete clinical records. An independent bulk cohort was obtained from the Therapeutically Applicable Research to Generate Effective Treatments (TARGET) osteosarcoma project (TARGET-OS; *n* = 87). For single-cell profiling, six osteosarcoma specimens (including pre-treatment biopsy and post-neoadjuvant surgical specimens) were collected and processed for scRNA-seq. This study was approved by the Ethics Committee of Peking University People's Hospital (No. 2024PHB432-001) and conducted in accordance with the Declaration of Helsinki. Written informed consent was obtained from all participants or their legal guardians.

### 2.2 Bulk RNA-Seq data processing and integration

Bulk RNA-seq generation, primary processing, and expression quantification for the institutional PKUPH-OS cohort followed procedures described previously. Publicly available TARGET-OS expression data and annotations were downloaded from the TARGET data portal (https://portal.gdc.cancer.gov/projects/TARGET-OS).

Before integration, gene identifiers were harmonized across datasets and genes with low information content were filtered by requiring FPKM >1 in at least 20% of samples within each dataset. Expression values were log₂-transformed [log₂(FPKM+1)] to a common scale suitable for pathway-level scoring. When multiple entries mapped to the same gene symbol, the entry with the highest mean expression was retained. The combined expression matrix was constructed using genes shared by both cohorts. To avoid circularity in using to-be-discovered subtype labels as a ComBat covariate, we used a two-pass iterative procedure following the standard recommended ComBat usage from the sva package (19). Pass 1: ComBat was applied to log₂(FPKM+1) values with cohort as the only batch term and no biological covariate, and an initial three-cluster consensus partition was derived from the resulting matrix. Pass 2: the Pass-1 partition was supplied as a protective biological covariate to preserve subtype-related variance, after which the final consensus clustering was performed. Pass-1 and Pass-2 cluster assignments agreed in 148 of 157 samples (94.3%); biological silhouette increased from 0.04 (Pass 0) to 0.21 (Pass 2) while cohort silhouette decreased from 0.79 to −0.005, indicating that the correction removed batch structure while preserving — not imposing — biological structure.

### 2.3 Pathway activity scoring and consensus clustering

Sample-level pathway activity profiles were computed using single-sample gene set enrichment analysis (20) (ssGSEA). Metabolism-relevant gene sets were manually curated from three canonical sources—KEGG metabolic pathways, Reactome metabolic pathways, and MSigDB Hallmark metabolic gene sets—and filtered to retain only those with 10–500 mapped genes in the integrated expression matrix. Pathway scores were standardized across samples (Z transformation within each pathway) prior to clustering. To address inter-database redundancy across KEGG, Reactome and Hallmark sources, a sensitivity analysis was performed in which 10 redundant pathways were removed and clustering was repeated on the resulting 14-pathway set; this recovered the identical three-class structure (adjusted Rand index = 0.94), confirming robustness to gene-set overlap.

Unsupervised subtype discovery was performed on the pathway score matrix using ConsensusClusterPlus (21). The consensus procedure used a maximum K of 8, 1,000 resampling iterations, 80% item subsampling per iteration, PAM (partitioning around medoids) clustering, and Pearson correlation-based distance (seed = 12,345). The number of clusters was selected based on concordant behavior across consensus matrices, CDF/delta area profiles, and silhouette structure.

To quantify stability of subtype assignments, several internal checks were applied (Supplementary Figure S1). These included silhouette width calculations using correlation-derived distances, bootstrap resampling with adjusted Rand index (ARI) benchmarking against the full-cohort solution, nearest template prediction (NTP) with cross-validation, and agreement across alternative clustering strategies applied to the same pathway score matrix. Parameter settings matched those used in the accompanying analysis pipeline.

### 2.4 Differential expression and functional enrichment analyses

Subtype-associated gene expression programs were examined in bulk RNA-seq using a one-vs.-rest design (each subtype contrasted against all remaining tumors). Differential expression testing was conducted using the limma R package (22) with empirical Bayes moderation (eBayes, trend = TRUE), with multiple testing controlled by the Benjamini–Hochberg procedure. Unless specified otherwise, genes meeting adjusted *P* < 0.05 and |log2 fold change| > 1 were considered differentially expressed.

For pathway-level interpretation, gene set enrichment analysis (GSEA) was performed (23) on pre-ranked gene lists derived from the differential expression statistics. Hallmark and KEGG gene sets from MSigDB served as primary references. Enrichment significance was evaluated using the fgsea R package with default permutation-based testing (gene set size: minSize = 15, maxSize = 500) and Benjamini–Hochberg FDR correction. Gene sets from MSigDB Hallmark (collection H) and KEGG_LEGACY (collection C2:CP:KEGG_LEGACY) were retrieved via the msigdbr R package.

Transcription factor (TF) activity was inferred using curated TF–target gene sets from MSigDB C3 regulatory target gene sets, specifically the TFT_LEGACY and GTRD subcollections (retrieved via the msigdbr R package, species = “Homo sapiens”). Per-sample TF activity scores were computed via enrichment-style scoring from bulk expression profiles and compared across subtypes with multiple-testing correction.

### 2.5 Tumor microenvironment estimation

Microenvironmental features were inferred from bulk RNA-seq using complementary signature-based approaches. Overall immune and stromal content was estimated with ESTIMATE (24), yielding immune-related and stromal-related scores. In parallel, immune cell representation was approximated by ssGSEA applied to published immune cell marker gene sets (Bindea-style signatures) (25). To provide an additional view with reduced sensitivity to rank-based effects, MCP-counter-like marker sets (26) (Becht-style) were also evaluated.

For immune checkpoint analysis, expression values of a prespecified checkpoint gene panel were extracted from normalized bulk expression matrices and compared across subtypes, with multiplicity handled by FDR correction. The checkpoint gene panel comprised ten genes: PDCD1 (PD-1), CD274 (PD-L1), PDCD1LG2 (PD-L2), CTLA4 (CTLA-4), LAG3 (LAG-3), HAVCR2 (TIM-3), TIGIT, VSIR (VISTA), ENTPD1 (CD39), and IDO1.

### 2.6 Single-cell RNA sequencing and analysis

Six osteosarcoma specimens were processed for scRNA-seq. Raw reads were aligned to GRCh38 and summarized into gene-by-cell count matrices using Cell Ranger. Downstream analyses were performed in Seurat (27) (v5.0) with SCTransform normalization; batch effects across specimens were mitigated with Harmony (28) (v1.0). Quality-control filters were applied as follows: cells with fewer than 200 detected genes (nFeature_RNA <200) or fewer than 500 UMIs (nCount_RNA <500) were excluded, and cells with mitochondrial transcript fraction exceeding 20% were removed; doublets were further filtered with DoubletFinder (29) using the expected multiplet rate from the 10x loading table. Cell clusters were annotated by canonical marker expression. Malignant cells were identified via inferCNV (v1.18; https://github.com/broadinstitute/inferCNV; analysis_mode = “subclusters”, cutoff = 0.1, denoise = TRUE, HMM = TRUE) using non-malignant immune populations (T cells, B cells and monocytes) as the diploid reference; cells within osteoblast (OB) and chondroblast (CB) compartments whose mean inferCNV score exceeded 1.5 standard deviations of the reference distribution were classified as malignant tumor cells. To project bulk-derived metabolic-subtype signatures onto single cells, the top 50 up-regulated DEGs per subtype (one-vs.-rest limma, ranked by t-statistic) were used as gene sets for Seurat's AddModuleScore function (control = 100 background genes, search = FALSE), yielding per-cell C1, C2 and C3 module scores. Following the recent benchmark of imputation and clustering methods for scRNA-seq data (30), which finds that imputation can improve downstream clustering and visualization, we used SCTransform normalization and AddModuleScore (100 control-gene bins) and elected to report results without imputation, given (i) the small number of signature genes used here (top 50 per subtype, with results dominated by high-expression members), (ii) the indirect effect of dropout on module-score projection compared to *de novo* clustering, and (iii) the robustness of our compartment-attribution conclusions to dropout (see top-25-gene check below). As a robustness check, recomputing C1/C2/C3 module scores using only the top-25 most-expressed genes of each signature yielded Spearman correlation >0.93 with the full-signature scores, indicating that compartment attribution is not driven by dropout-prone low-expression genes.

### 2.7 Statistical analysis

Clinical endpoints were defined before analysis. Overall survival (OS) was measured from diagnosis to death from any cause, with censoring at last contact for living patients. Progression-free survival (PFS) was measured from diagnosis to first documented progression/relapse or death, whichever occurred first, with censoring at last follow-up for event-free patients. Survival analyses were restricted to cases with available follow-up and endpoint information; patients lacking evaluable survival data were excluded from Kaplan–Meier and log-rank analyses. The data cut-off for survival follow-up in the PKUPH-OS cohort was 31 December 2024; for the TARGET-OS cohort, the most recent follow-up information available in the TARGET data portal at the time of analysis (last accessed 15 February 2025) was used. Continuous variables were compared using Wilcoxon rank-sum tests (two groups) or Kruskal–Wallis tests (more than two groups), unless distributional assumptions supported parametric alternatives. For single-cell module-score comparisons across cell types, Kruskal–Wallis omnibus tests were followed by pairwise Wilcoxon rank-sum tests with Benjamini–Hochberg correction; effect sizes were reported as Cliff's *δ*, with |*δ*| < 0.147 considered negligible, 0.147–0.33 small, 0.33–0.474 medium, and ≥0.474 large. Categorical variables were evaluated by chi-square test or Fisher's exact test as appropriate. Survival curves were estimated by the Kaplan–Meier method and compared by the log-rank test; multiple testing correction was performed using the Benjamini–Hochberg method. Unless otherwise specified, all tests were two-sided and a *P* < 0.05 was considered statistically significant; the directional vs-rest Wilcoxon procedure used in Methods §2.8 to derive subtype-specific drug recommendations is the one explicit exception, justified by its hypothesis-generating, one-sided framing (each subtype against the combined other two). A small number of patients with missing endpoint information were handled by complete-case analysis. To accommodate cross-cohort heterogeneity, analyses combining PKUPH-OS and TARGET-OS were performed in two ways: (i) pooled with cohort included either as a covariate or as a stratification factor in Cox regression; and (ii) separately within each cohort to confirm directional consistency of the subtype effect; both approaches yielded the same qualitative conclusions. Multivariable Cox regression used C2 as the reference subtype and was adjusted for age, sex and metastasis-at-diagnosis; for overall-survival models, Firth's penalized likelihood (31, 32) was additionally applied to address near-complete separation arising from the small number of OS events in C2.

### 2.8 Subtype-stratified treatment-response analysis

Predicted IC50 values for 198 anti-cancer agents were computed with the oncoPredict R package using the GDSC2 reference (33, 34); the top 32 targeted-therapy agents (small-molecule kinase, proteasome and epigenetic inhibitors) were retained for subtype comparison. Differential drug sensitivity across the three subtypes was assessed by Kruskal–Wallis test with Benjamini–Hochberg correction. Because the GDSC2 reference is trained on cancer cell lines and contains no representation of CAFs, MSCs or primary immune populations, predictions were post-filtered by mechanism-based compartment consistency: for each agent, its principal molecular target was annotated to a cellular compartment (C1 = stromal/mesenchymal; C2 = immune/mTOR; C3 = malignant/proliferation), and only agents whose oncoPredict “most-sensitive” subtype matched the annotated compartment were retained for biological interpretation (11 of 32 agents; the remaining 21 are listed in Figure 5E for transparency). Subtype-specific recommendations were further derived by one-sided Wilcoxon test of each subtype against the combined other two (vs-rest), with significance set at unadjusted *P* < 0.05. Post-neoadjuvant chemotherapy histologic response was assessed in the PKUPH cohort using percent tumor necrosis at definitive surgery (good responder: ≥90%; poor: <90%, Huvos grading).

### 2.9 Parsimonious gene panel for clinical detection

We trained a multinomial LASSO logistic regression (glmnet R package, alpha = 1, family = “multinomial”, 10-fold cross-validated) on the union of the top 50 up-regulated and top 50 down-regulated one-vs-rest DEGs per subtype (300 candidate gene slots in total; 150 unique gene symbols after across-subtype deduplication). The regularization parameter was selected to yield approximately 25 unique features distributed across all three classes. Classifier performance was evaluated by overall accuracy, Cohen's *κ* for agreement with the ssGSEA-derived subtypes, 5-fold cross-validated accuracy, and Kaplan–Meier survival stratification of panel-predicted subtypes.

## 3 Results

### 3.1 Osteosarcoma metabolic subtyping identifies cholesterogenic, redox-catabolic and OXPHOS-active states

An integrated bulk transcriptomic cohort was assembled by combining the institutional PKUPH-OS dataset (*n* = 70) and the public TARGET-OS dataset (*n* = 87), yielding 157 osteosarcoma tumors for metabolic-state discovery and downstream clinical association analyses. To minimize cohort-driven structure prior to subtype discovery, we inspected the global embedding of the merged dataset; samples from PKUPH and TARGET intermingled in the principal component space, arguing against overt cohort separation at the pathway-activity level (Supplementary Figure S1B).

**Figure 1:**
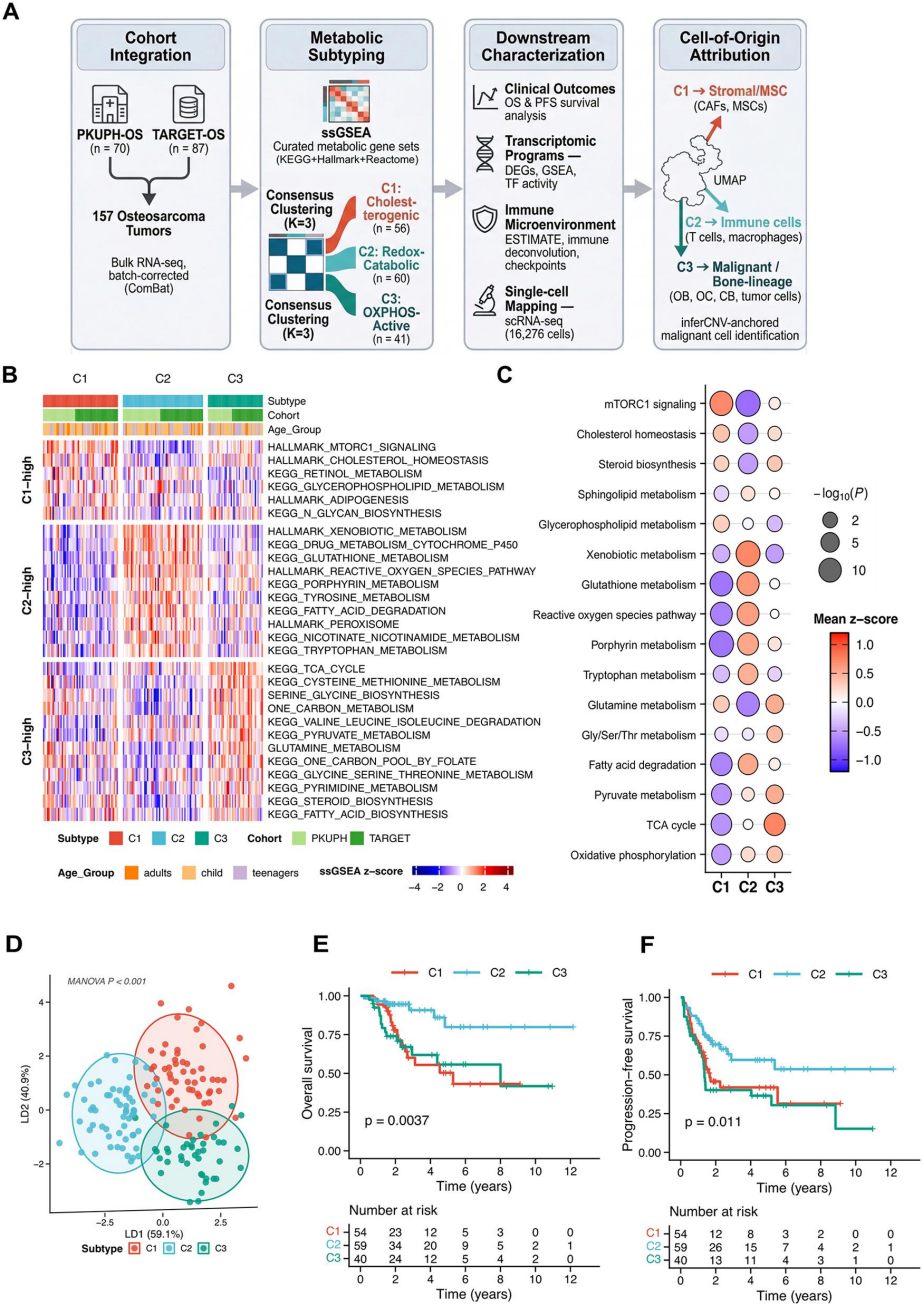
Pathway-level ssGSEA consensus clustering identifies three metabolic subtypes with distinct clinical outcomes in osteosarcoma. **(A)** Study workflow for metabolic subtyping. **(B)** Heatmap of ssGSEA pathway activity across tumors, grouped by the three consensus clusters (C1–C3). **(C)** Condensed module-level summary highlighting subtype-enriched metabolic programs. **(D)** Subtype assignment of the integrated cohort, partitioning tumors into C1 (Cholesterogenic), C2 (Redox-Catabolic), and C3 (OXPHOS-Active). **(E)** Kaplan–Meier overall survival (OS) curves comparing C1–C3. **(F)** Kaplan–Meier progression-free survival (PFS) curves comparing C1–C3. Risk tables indicate the numbers of patients with evaluable survival follow-up included in the Kaplan–Meier analyses; the integrated cohort contained 39 OS events (C1 = 18, C2 = 6, C3 = 15) and 76 PFS events (C1 = 29, C2 = 21, C3 = 26).

Subtype discovery was conducted on ssGSEA-derived metabolic pathway activity profiles using consensus clustering (Figure 1A). To select the most appropriate number of clusters, we evaluated K across multiple complementary stability criteria. Collectively, the consensus CDF behavior and its relative area-change suggested that *K* = 3 provided the most stable yet parsimonious solution (Supplementary Figures S1C, D), and the corresponding consensus matrix exhibited a well-demarcated block structure consistent with three coherent groups (Supplementary Figure S1E). This three-class solution was further supported by silhouette-based compactness/separation (Supplementary Figure S1F) and resampling stability (Supplementary Figure S1G), and it remained recoverable under an orthogonal template-based reclassification framework (Supplementary Figures S1H, I). Under *K* = 3, tumors were partitioned into C1 (*n* = 56), C2 (*n* = 60), and C3 (*n* = 41), forming the basis for downstream biological characterization (Supplementary Figure S1F; Figures 1B–D). Agreement across alternative clustering strategies further supported the robustness of the three-subtype solution (Supplementary Figure S1J).

We then interpreted each class by anchoring to the subtype-enriched pathway blocks in the ssGSEA heatmap and the condensed module-level summary (Figures 1B,C). Cholesterogenic (C1) showed a biosynthetic, membrane-oriented metabolic state, characterized by mTORC1-associated anabolic signaling coupled with cholesterol and complex lipid homeostasis, consistent with increased demand for membrane building and lipid remodeling (Figures 1B,C). Redox-Catabolic (C2) was dominated by a detoxification and redox-buffering program, together with enhanced substrate catabolism, indicating a state adapted to oxidative stress control and nutrient breakdown rather than macromolecular synthesis (Figures 1B, C). In contrast, OXPHOS-Active (C3) displayed a broad hypermetabolic configuration with prominent mitochondrial oxidative metabolism and coordinated biosynthetic support, including one-carbon and nucleotide-related processes, consistent with an energetically intensive state sustained by coupled precursor-supplying pathways (Figures 1B, C).

### 3.2 Clinical characteristics and prognostic relevance of metabolic subtypes in osteosarcoma

After defining three metabolic subtypes, we asked whether this classification captures clinically relevant differences in outcome. With a median follow-up of 2.7 years (IQR, 1.8–5.3), Kaplan–Meier curves separated the subtypes for both overall survival (OS; log-rank *P* = 0.0037) and progression-free survival (PFS; log-rank *P* = 0.011) (Figures 1E,F). In the integrated cohort with evaluable survival data (*n* = 153; C1 = 54, C2 = 59, C3 = 40 at-risk at baseline), the 3-year overall-survival landmark was 60.0% (95% CI 47.1–76.4%) in C1, 90.8% (95% CI 78.3–100.0%) in C2, and 61.9% (95% CI 47.6–80.4%) in C3, while 3-year PFS was 41.9% (95% CI 26.9–56.2%; 29 events) in C1, 59.7% (95% CI 43.3–72.7%; 21 events) in C2, and 40.2% (95% CI 24.8–55.2%; 26 events) in C3 (Figures 1E,F). Across the integrated cohort, 39 OS events (C1 = 18, C2 = 6, C3 = 15) and 76 PFS events (C1 = 29, C2 = 21, C3 = 26) were observed. The C2 favorable-risk pattern was already evident within PKUPH-OS alone, where only 2 OS events occurred among the 21 C2 patients. Because OS events were near-completely separated in C2 (2 events among 21 patients), Firth penalized likelihood was used in multivariable Cox regression to obtain non-degenerate hazard-ratio estimates rather than to recover statistical significance. Taken together, these patterns place C2 as the more favorable-risk metabolic state, whereas C1 and C3 align with higher-risk trajectories. Multivariable Cox regression confirmed that C1 and C3 retained significantly elevated overall-survival risk relative to C2 even after adjustment for age, sex and metastasis-at-diagnosis (Supplementary Figures S2A–D). Within the strict pediatric subgroup (≤14 yr, *n* = 81), overall survival remained significantly stratified across the three metabolic subtypes (log-rank *P* = 0.044) but progression-free survival did not (log-rank *P* = 0.32; 44 PFS events distributed across three subtypes). This non-significance is most parsimoniously attributable to the limited sample size of this subgroup rather than to an absence of subtype effect on PFS.

To address the possibility that outcome differences were attributable to baseline imbalance, we compared clinical characteristics across subtypes. Subtype distribution was comparable by dataset source (PKUPH vs. TARGET, *P* = 0.914) and by age (median age, *P* = 0.918; age-group distribution, *P* = 0.315). Given the limited granularity of public TARGET annotations, more detailed clinicopathologic comparisons were therefore examined primarily in the PKUPH cohort. Within PKUPH-OS, we did not observe significant subtype skew in age (median or categorical), sex, primary site, histology, chemotherapy, tumor necrosis rate, targeted therapy, or immunotherapy exposure (all *P* > 0.05; Table 1).

**Table 1:** Clinical characteristics of osteosarcoma metabolic subtypes in the PKUPH cohort.

Characteristic	All patients	C1	C2	C3	*P* value
No. of patients	70	30	21	19	
Age, years, median (IQR)	14.0 (11.2–16.0)	15.0 (11.2–16.0)	13.0 (12.0–16.0)	14.0 (11.5–15.0)	0.475
Age group, *n* (%)					0.609
Child (≤14 y)	37 (52.9%)	13 (43.3%)	13 (61.9%)	11 (57.9%)	
Teenagers (15–18 y)	22 (31.4%)	10 (33.3%)	6 (28.6%)	6 (31.6%)	
Adults (≥19 y)	11 (15.7%)	7 (23.3%)	2 (9.5%)	2 (10.5%)	
Sex, *n* (%)					0.098
Male	38 (54.3%)	21 (70.0%)	9 (42.9%)	8 (42.1%)	
Female	32 (45.7%)	9 (30.0%)	12 (57.1%)	11 (57.9%)	
Primary tumor site, *n* (%)					0.796
Femur	44 (62.9%)	19 (63.3%)	12 (57.1%)	13 (68.4%)	
Tibia	13 (18.6%)	5 (16.7%)	6 (28.6%)	2 (10.5%)	
Humerus	12 (17.1%)	5 (16.7%)	3 (14.3%)	4 (21.1%)	
Fibula	1 (1.4%)	1 (3.3%)	0 (0.0%)	0 (0.0%)	
Histologic subtype, *n* (%)					0.479
Conventional osteosarcoma	63 (90.0%)	27 (90.0%)	20 (95.2%)	16 (84.2%)	
Other subtypes	7 (10.0%)	3 (10.0%)	1 (4.8%)	3 (15.8%)	
Chemotherapy, *n* (%)					1.000
Yes	70 (100.0%)	30 (100.0%)	21 (100.0%)	19 (100.0%)	
No	0 (0.0%)	0 (0.0%)	0 (0.0%)	0 (0.0%)	
Tumor necrosis rate (%), median (IQR)	82.5 (56.8–95.2)	87.1 (64.8–97.1)	69.0 (20.6–84.5)	89.2 (62.9–94.6)	0.311
Targeted therapy, *n* (%)					0.578
Yes	15 (21.4%)	7 (23.3%)	3 (14.3%)	5 (26.3%)	
No	55 (78.6%)	23 (76.7%)	18 (85.7%)	14 (73.7%)	
Immunotherapy, *n* (%)					0.300
Yes	17 (24.3%)	10 (33.3%)	4 (19.0%)	3 (15.8%)	
No	53 (75.7%)	20 (66.7%)	17 (81.0%)	16 (84.2%)	

Data are presented as *n* (%) unless otherwise indicated. Age and tumor necrosis rate are shown as median (IQR). *P* values compare C1, C2, and C3; continuous variables were evaluated by Kruskal–Wallis test and categorical variables by chi-square test or Fisher's exact test, as appropriate. Tumor necrosis rate was assessable in 43 of 70 PKUPH-OS patients (those with post-neoadjuvant histology at definitive surgery); summary statistics for that variable are calculated on these 43 evaluable cases. IQR, interquartile range; PKUPH, Peking University People's Hospital.

To explore a parsimonious gene panel suitable for clinical detection, we trained a multinomial LASSO classifier on the union of subtype-specific DEGs (Methods 2.9) and swept panel sizes from 3 to 25 unique features. Smaller panels (3–10 genes) achieved only modest agreement with the ssGSEA-derived subtypes (Cohen's *κ* = 0.21–0.52), with C2 in particular requiring multiple immune-microenvironment-related genes to be robustly captured—reflecting that C2 is defined by tumor-microenvironment composition rather than by tumor-cell-intrinsic up-regulation. The smallest panel achieving substantial concordance (Cohen's *κ* = 0.67) and significant survival stratification (OS log-rank *P* = 2.6 × 10^−5^; PFS log-rank *P* = 3.2 × 10^−6^) required 25 genes (8/12/5 markers for C1/C2/C3, respectively; Supplementary Figure S3). With the currently available cohort size and the microenvironment-driven nature of the C2 niche, a strictly 3–5-gene panel cannot reach sufficient classification performance for clinical translation, and prospective panel design with larger cohorts will be required.

### 3.3 Transcriptomic pathway and regulatory differences among osteosarcoma metabolic subtypes

**Figure 2:**
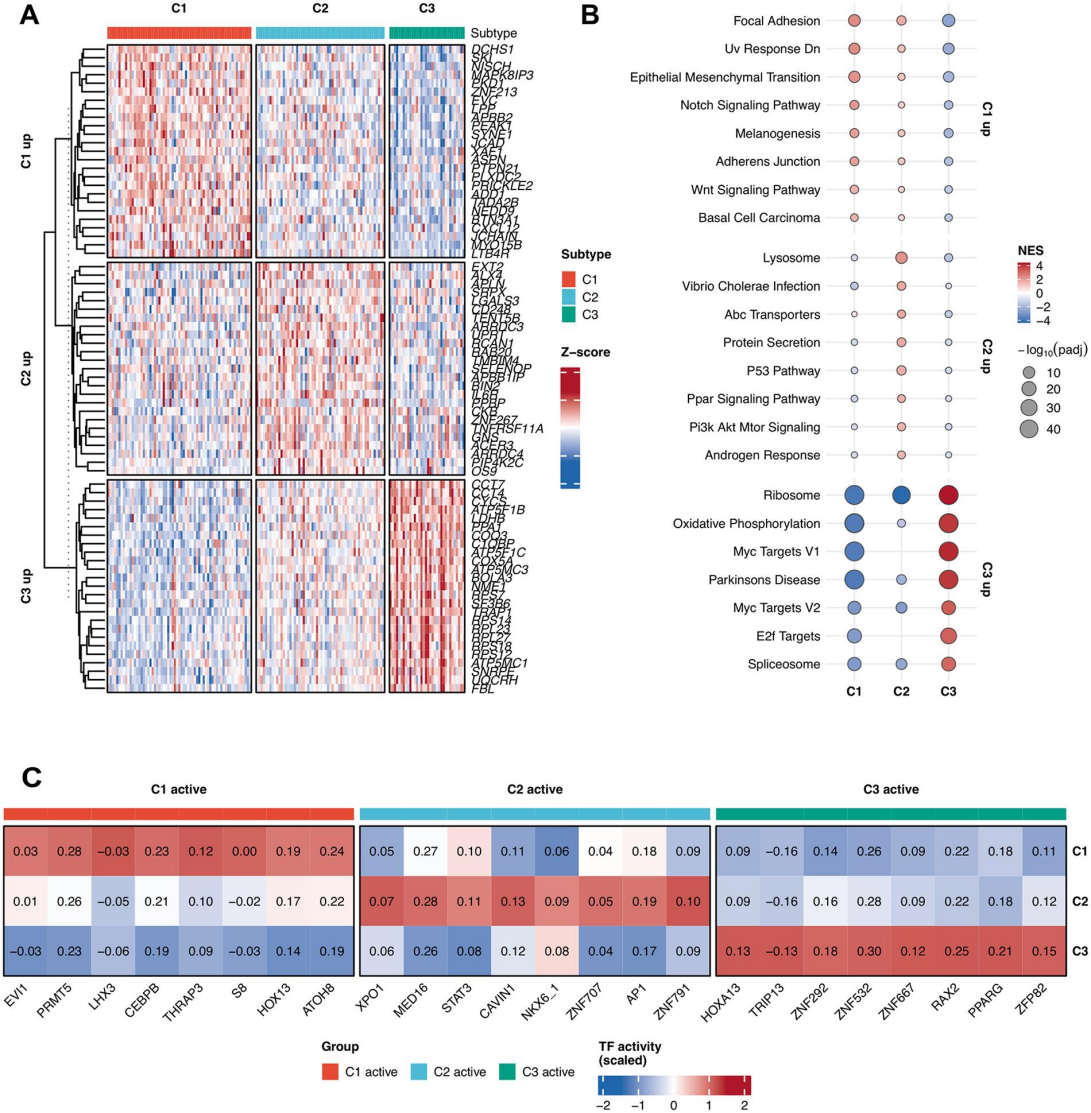
Distinct transcriptomic phenotypes and coordinated signaling programs characterize the three metabolic subtypes. **(A)** Heatmap of differentially expressed genes (DEGs) demonstrating robust subtype separation, with each class showing a compact, internally consistent upregulated gene set. **(B)** Gene set enrichment analysis (GSEA) summarizing subtype-specific pathway programs. **(C)** Inferred transcription factor (TF) activity highlights distinct regulatory wiring.

Bulk-level transcriptomics further resolved these three metabolic states into distinct gene-expression phenotypes and coordinated pathway programs (Figures 2A, B). The DEG heatmap showed broad subtype separation, with each class marked by a compact, internally consistent set of upregulated transcripts (Figure 2A). C1 was dominated by a stromal/adhesion-leaning expression pattern, exemplified by genes such as DCHS1, SYNE1, PEAK1, and ASPN, whereas C2 preferentially upregulated immune- and stress-interface genes including LGALS3, CD248, IL6R, and SELENOP; in contrast, C3 was characterized by elevated mitochondrial and translational machinery, with prominent OXPHOS complex components and ribosomal genes (e.g., ATP5F1B, COX5A, CYCS, RPS7, RPL23) (Figure 2A). At the pathway level, GSEA indicated that these differences were not isolated gene effects but reflected subtype-specific, concerted programs (Figure 2B): C1 enriched mesenchymal and invasion-associated signaling (Focal Adhesion, EMT, Wnt, Notch), C2 enriched regulated/catabolic modules (P53 Pathway, PPAR Signaling, Lysosome, Protein Secretion), and C3 concentrated a coupled “OXPHOS–proliferation” axis (Oxidative Phosphorylation, Ribosome, MYC Targets V1/V2, E2F Targets) (Figure 2B). Upstream regulatory inference provided additional evidence that the three metabolic states were accompanied by distinct regulatory landscapes (Figure 2C). In C1, the dominant inferred regulators included EVI1, PRMT5, LHX3, CEBPB, THRAP3, S8, HOX13, and ATOH8. C2 displayed a different activity pattern, characterized by XPO1, MED16, STAT3, CAVIN1, NKX6_1, ZNF707, AP1, and ZNF791. In contrast, C3 was defined by HOXA13, TRIP13, ZNF292, ZNF532, ZNF667, RAX2, PPARG, and ZFP82 (Figure 2C). Rather than converging on the same upstream regulators across subtypes, these results support the view that each metabolic class is coupled to a distinct transcriptional-control context, further separating the three states at the regulatory layer.

### 3.4 Immune microenvironment heterogeneity across osteosarcoma metabolic subtypes

**Figure 3:**
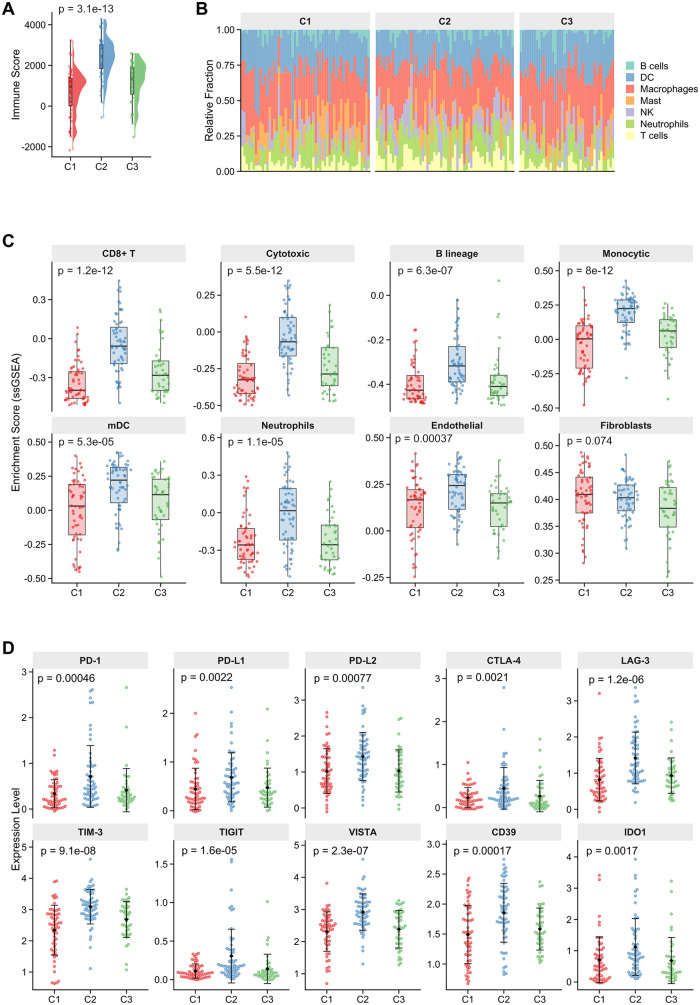
Immune microenvironment heterogeneity across osteosarcoma metabolic subtypes. **(A)** ESTIMATE Immune Score across C1–C3 with C2 exhibiting the highest immune infiltration. **(B)** ssGSEA-based relative immune-cell composition across subtype. **(C)** MCP-counter–like absolute abundance scores for immune and stromal lineages. **(D)** Expression of immune checkpoint transcripts across subtypes.

Consistent with the subtype-divergent programs described above, the tumor immune microenvironment differed markedly across C1–C3. ESTIMATE revealed a strong subtype effect on immune content (Immune Score, Kruskal–Wallis *P* = 3.1 × 10^−13^), with C2 showing the highest immune infiltration (Figure 3A). In ssGSEA-based relative composition, macrophages constituted the dominant fraction across all groups, whereas the relative contributions of T cells and dendritic cells shifted in a subtype-dependent manner, giving C2 a distinct “immune fingerprint” (Figure 3B). Moving from composition to absolute abundance, MCP-counter–like scores indicated that most immune and stromal lineages were significantly increased in C2, and the clearest differences were seen in adaptive effector signals—CD8+ T cells (*P* = 1.2 × 10^−12^) and cytotoxic lymphocytes (*P* = 5.5 × 10^−12^)—together with myeloid compartments such as monocytic lineage cells (*P* = 8 × 10^−12^) and myeloid dendritic cells (*P* = 5.3 × 10^−5^) (Figure 3C). Notably, fibroblasts were the only population that did not reach significance (*P* = 0.074) (Figure 3C). This immune-inflamed background in C2 was paralleled by a coordinated upregulation of multiple checkpoint transcripts, exemplified by TIM-3 (*P* = 9.1 × 10^−8^), TIGIT (*P* = 1.6 × 10^−5^), PD-1 (*P* = 4.6 × 10^−4^), and CTLA-4 (*P* = 2.1 × 10^−3^) (Figure 3D), supporting the notion that immune activation and compensatory inhibitory signaling co-exist within the C2 subtype.

### 3.5 Single-cell mapping delineates the cellular origins and localization of osteosarcoma metabolic subtype signatures

**Figure 4:**
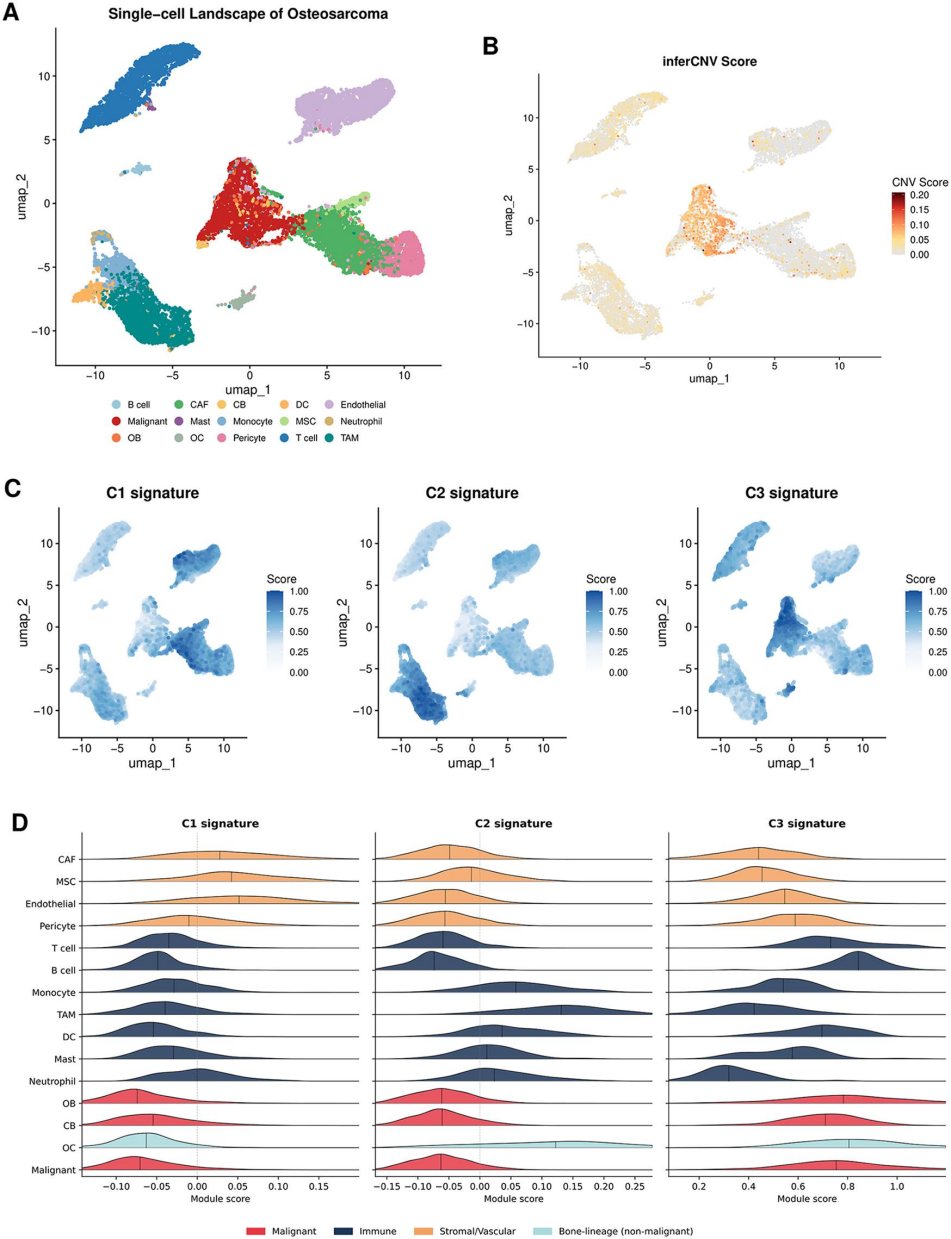
Single-cell mapping delineates the cellular origins and localization of bulk metabolic subtype signatures. **(A)** Single-cell atlas (16,276 cells) defining major cellular compartments, including immune lineages (T cells, B cells, monocyte/macrophage lineage, dendritic cells, mast cells, neutrophils), stromal/vascular-associated populations (CAFs, MSCs, endothelial cells, pericytes), and bone microenvironment–characteristic lineages (osteoblasts, osteoclasts, chondroblasts). **(B)** inferCNV-based copy-number inference using non-malignant immune cells as diploid reference to support malignant-cell identification. **(C)** Projection of bulk-derived C1–C3 metabolic signatures onto the single-cell atlas to visualize cell-type–resolved distribution patterns. **(D)** Per-cell module scores for the C1, C2 and C3 signatures, stratified by annotated cell type and coloured by cellular compartment (stromal/vascular = orange; immune = dark blue; bone-lineage = light blue; malignant = red). Between-cell-type module-score differences were tested by Kruskal–Wallis followed by pairwise Wilcoxon rank-sum tests with Benjamini–Hochberg correction; effect sizes are reported as Cliff's *δ*. Kruskal–Wallis omnibus tests were highly significant for each signature (all *P* < 1 × 10^−200^). At the compartment level, anchor-vs-malignant contrasts reach BH-adjusted *P* < 1 × 10^−10^ with Cliff's *δ* in the medium-to-large range (+0.41 to +0.80). At the cell-type level, anchor-vs-non-anchor contrasts for each signature (CAF and MSC for C1; TAM, Monocyte and DC for C2; Malignant for C3) all reached BH-adjusted *P* < 1 × 10^−100^ with Cliff's *δ* in the medium-to-large range (+0.65 to +0.96). Statistical annotations are omitted from the figure for clarity.

Because bulk RNA-seq inherently captures a composite transcriptomic signal from multiple cellular constituents within tumor tissue, we next leveraged single-cell data to trace the cellular sources of the C1–C3 metabolic signatures. Using a single-cell atlas comprising 16,276 cells, we first established a comprehensive cellular taxonomy. Immune compartments encompassed T cells, B cells, the monocyte/macrophage lineage, dendritic cells (DCs), mast cells, and neutrophils, whereas stromal and vascular-associated populations included cancer-associated fibroblasts (CAFs), mesenchymal stromal cells (MSCs), as well as endothelial cells and pericytes. In addition, we resolved bone microenvironment–characteristic lineages, including OB (osteoblasts), OC (osteoclasts), and CB (chondroblasts) (Figure 4A). We then performed inferCNV-based copy-number inference using non-malignant immune cells as a diploid reference. Specifically, we designated a subset of cells within the OB and CB compartments that exhibited higher inferCNV signals as malignant tumor cells, thereby providing an expression-supported “malignancy anchor” for subsequent signature projection (Figure 4B).

Projection of the bulk-derived metabolic signatures onto the single-cell atlas revealed a clear and complementary pattern of cellular localization (Figures 4C, D). The C1 signature preferentially scored in stromal-associated populations, including CAFs and MSCs, suggesting that the bulk C1 phenotype is more strongly shaped by mesenchymal contributions (Figure 4D). In contrast, the C2 signature was primarily enriched in immune cell populations, aligning with the immune-upregulated profile observed at the bulk level (Figure 4D). The C3 signature mapped predominantly to bone-lineage populations (OB/OC/CB) and malignant cells (Figure 4D), consistent with a more tumor-intrinsic and bone-lineage–linked metabolic program. Notably, these patterns were not uniform within broad compartments: within immune cells, C2 mapping displayed evident cell-type preference, and within bone-lineage populations, C3 broadly covered OB/OC/CB yet reached its highest mapping intensity in malignant cells, underscoring cell type–resolved heterogeneity in signature distribution (Figure 4D). To verify that this attribution is not driven by tumor-microenvironment composition, we re-scored each cell against the three signatures within the inferCNV-anchored malignant subset alone. Among 2,472 inferCNV-defined malignant cells, 2,469 (99.88%) scored highest on the C3 module, 3 (0.12%, all from specimen S3-Post) scored highest on C1, and none scored highest on C2, supporting C3 as a tumor-cell-intrinsic OXPHOS program (Supplementary Figure S4).

### 3.6 Subtype-stratified treatment-response analysis identifies actionable targeted-therapy vulnerabilities

**Figure 5:**
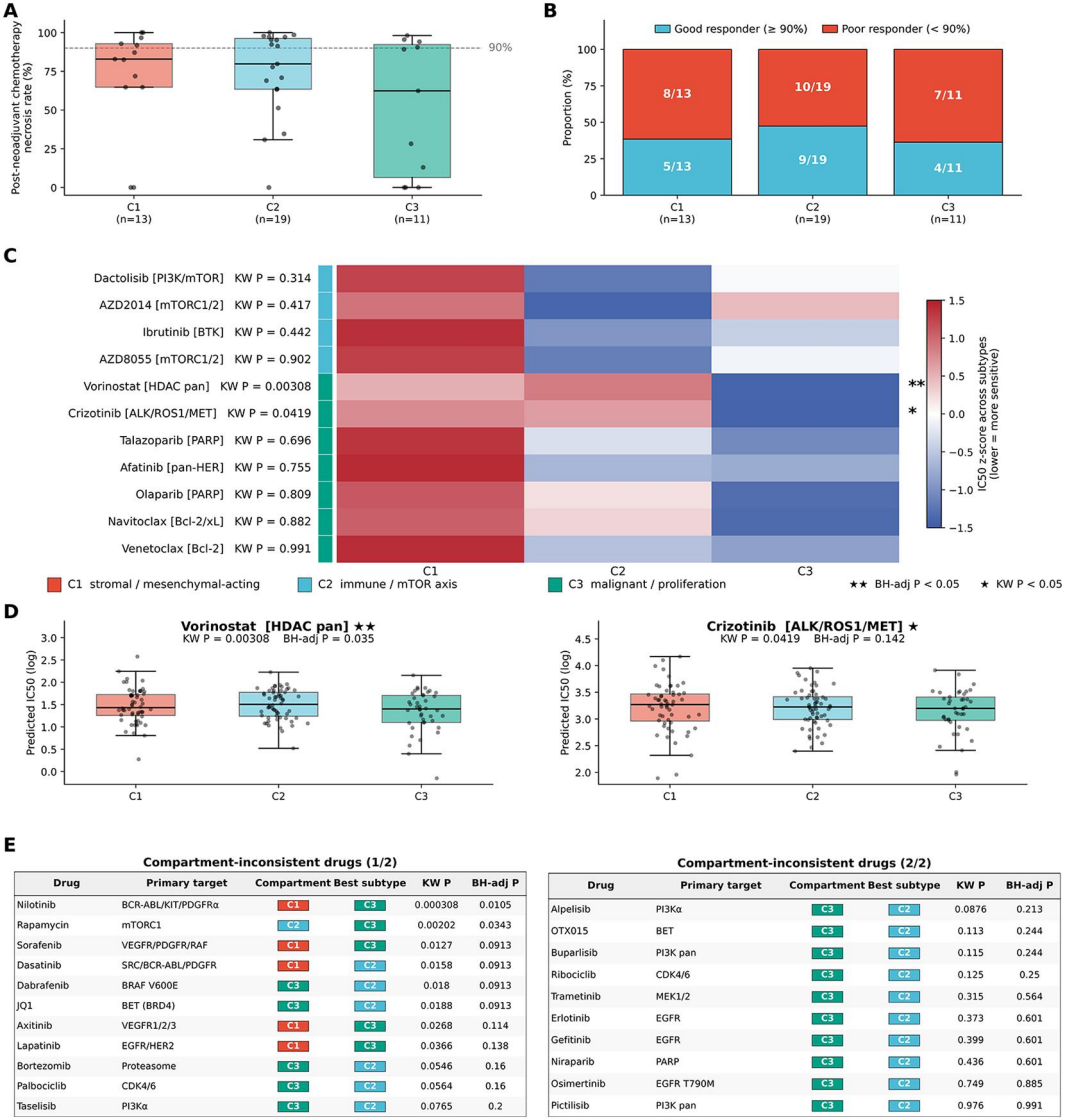
Subtype-stratified treatment-response analysis. **(A)** Post-neoadjuvant-chemotherapy necrosis rate by subtype in the PKUPH cohort (*n* = 43); Kruskal–Wallis test. **(B)** Stacked bar of good (≥90% necrosis) vs. poor (< 90%) responder rates; Fisher exact test. **(C)** Heatmap of predicted IC50 (oncoPredict, GDSC2; z-scored across subtypes within each drug) for the 11 KW-tested agents that passed the compartment-consistency filter; row labels list each drug's primary target and Kruskal–Wallis P, and a left-side colour strip indicates the annotated compartment (C1 stromal/mesenchymal-acting, C2 immune/mTOR axis, C3 malignant/proliferation); “★★” marks BH-adjusted *P* < 0.05, “★” marks unadjusted KW *P* < 0.05. **(D)** Per-drug predicted IC50 (log) boxplots for the two top-ranking compartment-consistent agents, Vorinostat (HDAC pan; KW *P* = 0.003, BH *P* = 0.035) and Crizotinib (ALK/ROS1/MET; KW *P* = 0.042, BH *P* = 0.142). **(E)** Tabular listing of the 21 agents excluded by the compartment-consistency filter, showing the annotated compartment, the oncoPredict best subtype, and KW/BH-adjusted *P*-values.

We further explored whether each subtype harbors distinct therapeutic vulnerabilities at two levels (Figure 5). Within the PKUPH cohort, post-neoadjuvant chemotherapy response did not differ significantly across subtypes (Figures 5A, B), although C3 displayed the widest response distribution, suggesting a chemo-refractory subset within this subtype. We complemented the clinical analysis with in-silico pharmacogenomic association using oncoPredict against the GDSC2 reference. Because GDSC2 is trained on cancer cell lines and contains no representation of CAFs, MSCs, or primary immune populations, oncoPredict outputs are best interpreted as bulk-transcriptome-level pharmacogenomic associations rather than as malignant-cell-intrinsic sensitivities. For theoretical consistency, we therefore refined Figure 5 to retain only drug predictions whose principal cellular target is compatible with each subtype's dominant compartment: C1 (stromal/mesenchymal-acting agents), C2 (immune- or mTOR-axis agents that act on TAMs and activated T cells), and C3 (mitochondrial/OXPHOS-targeting and proliferation-targeting agents, retained with a tumor-cell-intrinsic interpretation supported by the single-cell evidence above). Of 32 KW-tested targeted drugs, 11 satisfied this compartment-consistency filter; 21 predictions in which the oncoPredict-best subtype was incompatible with the mechanism-based compartment were removed (Figure 5C). Within the compartment-consistent set, the only BH-significant association was Vorinostat (HDAC pan; KW *P* = 0.003, BH *P* = 0.035) with C3-favored sensitivity (Figure 5D, left panel); Crizotinib (ALK/ROS1/MET) showed a trend-level signal (KW *P* = 0.042, BH *P* = 0.14; Figure 5D, right panel).

## 4 Discussion

Using osteosarcoma bulk transcriptomes, we treated pathway-level ssGSEA phenotypes as the input space for unsupervised consensus clustering and identified three stable, biologically interpretable metabolic subtypes. Cholesterogenic C1 was marked by elevated cholesterol homeostasis and membrane lipid–related metabolism; Redox-Catabolic C2 was characterized by upregulated redox detoxification modules (xenobiotic metabolism and GSH/ROS programs) together with enhanced catabolic routes (including fatty acid degradation); and OXPHOS-Active C3 featured strengthened TCA–OXPHOS activity accompanied by active precursor-supporting metabolism such as one-carbon and nucleotide-related processes. Clinically, these classes stratified outcome in a coherent manner: C2 aligned with the most favorable overall survival and progression-free survival, whereas C1 and C3 tracked with relatively adverse trajectories. By integrating single-cell data for cellular-source attribution, we further observed that the C1 metabolic signature was preferentially expressed in stromal/mesenchymal populations (e.g., CAFs and MSCs), the C2 signature mapped predominantly to immune compartments (e.g., TAMs, monocytes, and dendritic cells), and the C3 signature was concentrated within malignant tumor cells. This mapping argues that bulk-derived metabolic subtyping, to a substantial extent, captures differences in tumor-microenvironment composition and the ecological state of the tissue niche that those compartments collectively establish. In that sense, the current taxonomy can function both as a risk-stratification tool and as a structured framework for parsing tissue-level biological variation in osteosarcoma. Throughout this work, we use the term “metabolic niche state” alongside “metabolic subtype” to emphasize that bulk-derived phenotypes reflect coordinated programs of multiple coexisting cell populations rather than tumor-cell-intrinsic on/off settings — an interpretation directly supported by the single-cell cellular-source attribution presented above. Cellular-composition effects on bulk transcriptomic signatures are well recognized (35, 36); the contribution of the present study is to make this attribution explicit specifically for osteosarcoma metabolic subtyping and to translate it into clinically actionable framing of subtype-targeted strategies.

A closer look at C1 suggests that its biology cannot be reduced to a superficial label of “upregulated cholesterol synthesis.” More consequential is the apparent co-directionality between its membrane/lipid metabolic program and invasion-related signaling networks—a resonance-like coupling that is hard to ignore. Clinically, C1 patients followed a less favorable survival trajectory, implying that this state is unlikely to be a neutral accompaniment and may instead interlock with tissue programs that facilitate osteosarcoma progression. Mechanistically, the metabolic backbone of this subtype is more parsimoniously interpreted as coordinated activation of mTORC1 signaling together with cholesterol homeostasis (37, 38) and glycerophospholipid metabolism, rather than as evidence for a specific upstream transcription-factor-defined program. In parallel, coordinated enrichment of EMT, Wnt/Notch, and focal adhesion programs—each tied to migration, adhesion remodeling, and mesenchymal transition—positions C1 as a tissue state organized around membrane lipid supply and structural reconfiguration. Biologically, changes in cholesterol and membrane-lipid composition can, in principle, influence membrane fluidity (39, 40), receptor or signaling-complex assembly, and cytoskeletal coupling, thereby furnishing a material substrate for migratory and invasive phenotypes; within the evidentiary bounds of our data, however, this relationship is best framed as consistent with—rather than proving—a direct causal chain. Importantly, single-cell attribution indicates that the C1 signature preferentially originates from stromal-associated cells (including CAFs and MSCs), which helps interpret the prognostic signal: the adverse course of C1 may reflect a stromal-driven metabolic–signaling program that aligns with an invasion-permissive niche, rather than a purely tumor-cell-intrinsic metabolic preference. Bone-tropic nanomaterial delivery systems (41) offer a complementary translational route for reaching mesenchymal-niche-driven vulnerabilities at primary and metastatic osteosarcoma sites, providing a candidate scaffold for C1-directed stromal-modulating strategies.

C2 (Redox-Catabolic) presented an unusual triad in osteosarcoma—immune engagement coupled to catabolic metabolism and detoxification/antioxidant circuitry—and this architecture cohered with its favorable prognosis. Rather than anchoring this subtype to a specific transcription-factor-centered regulatory model, the present data support a more conservative interpretation based on the convergence of immune-cell enrichment, immune-pathway activation, and redox/catabolic adaptation within the tissue microenvironment. Functionally, the co-expression of immune pathways and checkpoint-related signals suggests a state that is (42, 43) “immune-active yet regulated,” closer to a dynamic balance under activation with concurrent exhaustion pressure than to a static condition of uniformly high immunity. Running alongside this immune context, C2 was distinguished by stronger xenobiotic metabolism, GSH/ROS detoxification, and multiple catabolic routes (including fatty acid degradation), resembling a tissue-level “stress-buffering system” that accommodates oxidative stress and drug/endogenous toxic burdens. The joint presence of reduced mTORC1 signaling and strengthened p53-pathway activity adds a second layer of interpretation: rather than equating C2 with simple energy deprivation, the data fit an adaptive state in which, under inflammatory and immune-activation pressures, tissue restrains anabolic drive while augmenting clearance and breakdown to maintain homeostasis. Even so, detoxification and GSH systems carry a well-known double-edged character in therapeutic contexts: in some settings, they may increase resistance to chemotherapy-induced ROS, whereas in others they may preserve immune-cell function and sustain effector activity. Because our inference is primarily transcriptional and correlational at the tissue-composition level, we interpret C2 most conservatively as an ecological niche with higher immune participation and enhanced capacity to manage oxidative/toxic stress, with its favorable outcomes plausibly reflecting microenvironmental immune advantage. The simultaneous up-regulation of multiple immune-checkpoint transcripts (TIM-3, TIGIT, PD-1, CTLA-4) within an otherwise immune-active and favorable-prognosis subtype warrants explicit interpretation. We propose that C2 represents an “immune-active-but-regulated” state in which sustained antigen exposure drives compensatory inhibitory signaling — a configuration where checkpoint expression marks effector engagement rather than terminal exhaustion. This interpretation is consistent with the broadly increased CD8⁺ T-cell and cytotoxic-lymphocyte signals (MCP-counter, Figure 3C) and with the favorable C2 outcome trajectory; importantly, it identifies C2 as the subtype most likely to benefit from checkpoint-blockade therapy, an actionable hypothesis to be tested in future trials. At the transcription-factor level (Figure 2C), several of the data-driven top TFs do have established cancer-metabolism or immune-signalling roles that align coherently with each subtype's biology: in C1, PRMT5 (an arginine methyltransferase implicated in lipid/cholesterol-pathway regulation in cancer) and CEBPB (a mesenchymal-lineage and osteogenic transcription factor) are concordant with C1's cholesterol/membrane-lipid programme; in C2, STAT3 is the most directly interpretable hit, given its canonical role in IL-6/JAK–STAT immune signalling that is consistent with the immune-engaged C2 phenotype, together with XPO1 as a validated cancer drug target; in C3, TRIP13 (a mitotic checkpoint regulator and established oncogene) and PPARG (a nuclear receptor capable of driving mitochondrial biogenesis) align with C3's coupled OXPHOS–proliferation programme. We recognise that several of the remaining top TFs (ZNF- and HOX-family members) have not been functionally characterised in osteosarcoma metabolism and we present these as hypothesis-generating candidates for follow-up rather than validated regulators. Conceptually, the C1 lipid-anabolic state is parsimoniously read against a sterol/isoprenoid-biosynthesis axis (e.g., SREBF1/SREBF2), the C2 catabolic/redox-buffering state against lysosome–autophagy regulators of the CLEAR network (e.g., TFEB, FOXO3), and the C3 OXPHOS-active state against mitochondrial biogenesis co-activators (e.g., MYC, NRF1, TFAM); these canonical regulators do not themselves appear among the top-ranked data-driven TFs in Figure 2C, indicating that the data-driven list is complementary to — not a re-discovery of — the canonical regulatory landscape. A recent two-sample Mendelian randomization analysis identified putatively causal contributions of several circulating immune cell traits to osteosarcoma risk (44), raising the possibility that part of the C2 vs. C3 immune-composition divergence reflects host germline immune set-points rather than purely tumor-driven recruitment; disentangling these contributions will require paired germline–immune profiling in future cohorts.

Distinct from the more “niche-signal”–leaning profiles of C1 and C2, C3 (OXPHOS-Active) exhibited a more overt mitochondrial-centric metabolic architecture. Elevated TCA cycle and oxidative phosphorylation, together with concurrent upregulation of Ser–Gly/one-carbon metabolism, pyrimidine metabolism, and multiple amino-acid catabolic pathways, converged on a tightly coupled state of “mitochondrial energy supply–precursor provisioning–biosynthesis/proliferation”. At the systems level, this pattern is more directly supported by the coordinated enrichment of energy-production and biosynthetic programs than by evidence for any single dominant upstream transcriptional regulator. Accompanying this high-OXPHOS configuration was an immune-desert phenotype, with globally weaker immune signals, suggesting limited effective immune infiltration or an immune-excluded state within the tissue microenvironment. While the present data cannot establish causality, conceptually plausible mechanisms include high oxygen consumption and substrate competition by tumor cells, along with metabolism-linked hypoxia shaping, each of which could impair T-cell function and promote immune exclusion. Crucially, single-cell attribution mapped the C3 signature predominantly to malignant tumor cells, which raises confidence in the interpretation: at least at the level of cellular provenance, C3 more closely reflects tumor-cell metabolic rewiring rather than being primarily driven by shifts in non-malignant TME composition. Mechanistically, the immune-excluded phenotype of C3 (45, 46) is most parsimoniously interpreted as a downstream consequence of high mitochondrial oxygen consumption and substrate competition by malignant cells: locally depleted glucose, fatty-acid intermediates and amino acids, together with hypoxia-driven adenosinergic and lactate signaling, can compromise effector T-cell metabolic fitness and recruitment. Our single-cell verification (Supplementary Figure S4) — in which 2,469 of 2,472 inferCNV-defined malignant cells (99.88%) score highest on C3 even when scored only against tumor cells — supports the view that C3 reflects a tumor-cell-intrinsic energetic program; the immune-cold phenotype is therefore best framed as a metabolic exclusion mechanism rather than a primary immunological defect. Although our data cannot directly establish causality, this framework generates testable predictions: blocking lactate export, adenosine signaling or OXPHOS-derived hypoxic niches should partially restore immune infiltration in C3 tumors.

One apparent paradox emerged from the bulk–single-cell integration. Although bulk transcriptomes separated tumors into three stable metabolic subtypes, single-cell mapping suggested that malignant cells were highly concentrated—almost “singly assigned”—to one metabolic identity, with C1 and C2 bulk signals largely carried by stromal and immune compartments, respectively, and C3 aligning most closely with malignant tumor cells. At a higher interpretive level, this observation is consistent with the established understanding that bulk classifiers reflect coordinated cellular composition: a bulk “metabolic subtype” in our analysis is most accurately read as a tissue-niche subtype, capturing the metabolic landscape shaped jointly by coexisting cellular communities, rather than representing three intrinsic on/off settings inside tumor cells. Under this niche-oriented framing, the favorable outcomes of C2 may partially reflect the abundance and functional state of immune components; the adverse outcomes of C1 may track with a stroma-driven invasive niche; and the adverse course of C3 is more plausibly rooted in tumor-cell-intrinsic coupling between mitochondrial energy production, biosynthetic support, and proliferative programs. Beyond osteosarcoma, this structure carries a methodological implication: without explicit cellular-source attribution, bulk metabolic conclusions can easily misread TME metabolic activity as tumor-cell rewiring, which in turn can misdirect mechanistic hypotheses and downstream target selection. For that reason, we view cell-of-origin attribution as a necessary step for future bulk-based metabolic subtyping in osteosarcoma and, more broadly, in other solid tumors, so that biological interpretation and translational extrapolation rest on evidence that better approximates true tissue architecture.

Several limitations warrant careful consideration. First, although we integrated a public cohort with an institutional cohort and applied formal ComBat batch-effect correction, the overall design remains retrospective; between-cohort differences in clinical management, sample handling, and residual unmodelled batch effects cannot be fully eliminated, and the association between metabolic subtypes and outcome is best interpreted as a stable statistical signal suitable for risk stratification rather than a direct causal pathway. Second, our characterization of “metabolic activity” relies primarily on pathway-level scoring (ssGSEA) and related computational inference from bulk transcriptomes; transcript abundance is an indirect proxy for actual metabolic flux and cannot substitute for direct measurements of metabolite concentrations or pathway turnover. Mechanistic claims should therefore be regarded as transcriptionally grounded hypotheses rather than verified flux statements. Third, the single-cell analysis provided key evidence for cellular-source attribution, but the scRNA-seq cohort comprised only six specimens, and these inferences should be replicated in larger single-cell osteosarcoma atlases as they become available. Fourth, public osteosarcoma transcriptomic cohorts remain very limited, and a sufficiently powered external validation dataset is currently lacking; future work should pursue independent external validation as additional cohorts become available. Fifth, the study is largely computational and lacks direct functional validation of the metabolic axes defining C1/C2/C3; we therefore present several mechanistic inferences as testable hypotheses with explicit prioritization for follow-up work. Sixth, osteosarcoma is a rare malignancy and very few publicly available cohorts offer sufficient size and clinical-annotation granularity; ComBat aligns expression distributions but does not — and is not intended to — correct heterogeneity in follow-up duration, treatment era, or endpoint definition between PKUPH (June 2012 to December 2023, median follow-up 2.7 years; data cut-off 31 December 2024) and TARGET (median follow-up 5.6 years from TARGET-OS portal annotations), and assembling a more clinically homogeneous combined cohort is not feasible within the timeline of this revision. Seventh, our compartment attribution is based on module-score projection at the cell-type level and does not employ recently developed machine-learning frameworks that reconstruct spatial cellular distributions from dissociated scRNA-seq (47); integration with dedicated spatial transcriptomic data is a clear next step.

## 5 Conclusions

In summary, by quantifying pathway-level metabolic phenotypes from osteosarcoma bulk transcriptomes and applying consensus clustering, we delineated three stable and interpretable metabolic subtypes with clear clinical stratification: C2 (Redox-Catabolic) aligned with the most favorable outcomes, whereas C1 (Cholesterogenic) and C3 (OXPHOS-Active) tracked with less favorable survival courses. Cell-of-origin attribution using single-cell data further clarified that bulk-level metabolic differences correspond, to a substantial degree, to distinct tumor-microenvironment niches and their dominant cell populations—C1 signatures largely carried by stromal/mesenchymal cells, C2 signatures largely carried by immune cells, and C3 signatures aligning more closely with malignant tumor-cell intrinsic metabolic state. This work therefore offers both a practical framework for osteosarcoma risk stratification and a more interpretable “metabolic niche” perspective that can guide functional validation and translational exploration of subtype-specific vulnerabilities and microenvironmental interactions.

## Data Availability

Publicly available datasets were analyzed in this study. This data can be found here: The sequencing data reported in this study have been deposited in the Gene Expression Omnibus (GEO) database of the National Center for Biotechnology Information (NCBI) under accession number GSE317512. The TARGET-OS dataset is publicly available at https://portal.gdc.cancer.gov/projects/TARGET-OS.
