# Supplementary figures and images for: Transcriptome-Inferred metabolic subtypes define prognostic and immune ecosystems in osteosarcoma at single-cell resolution
Source: Front Pediatr. 2026 Jun 18;14:1831516. doi: 10.3389/fped.2026.1831516 (PMC13323242; doi:10.3389/fped.2026.1831516)

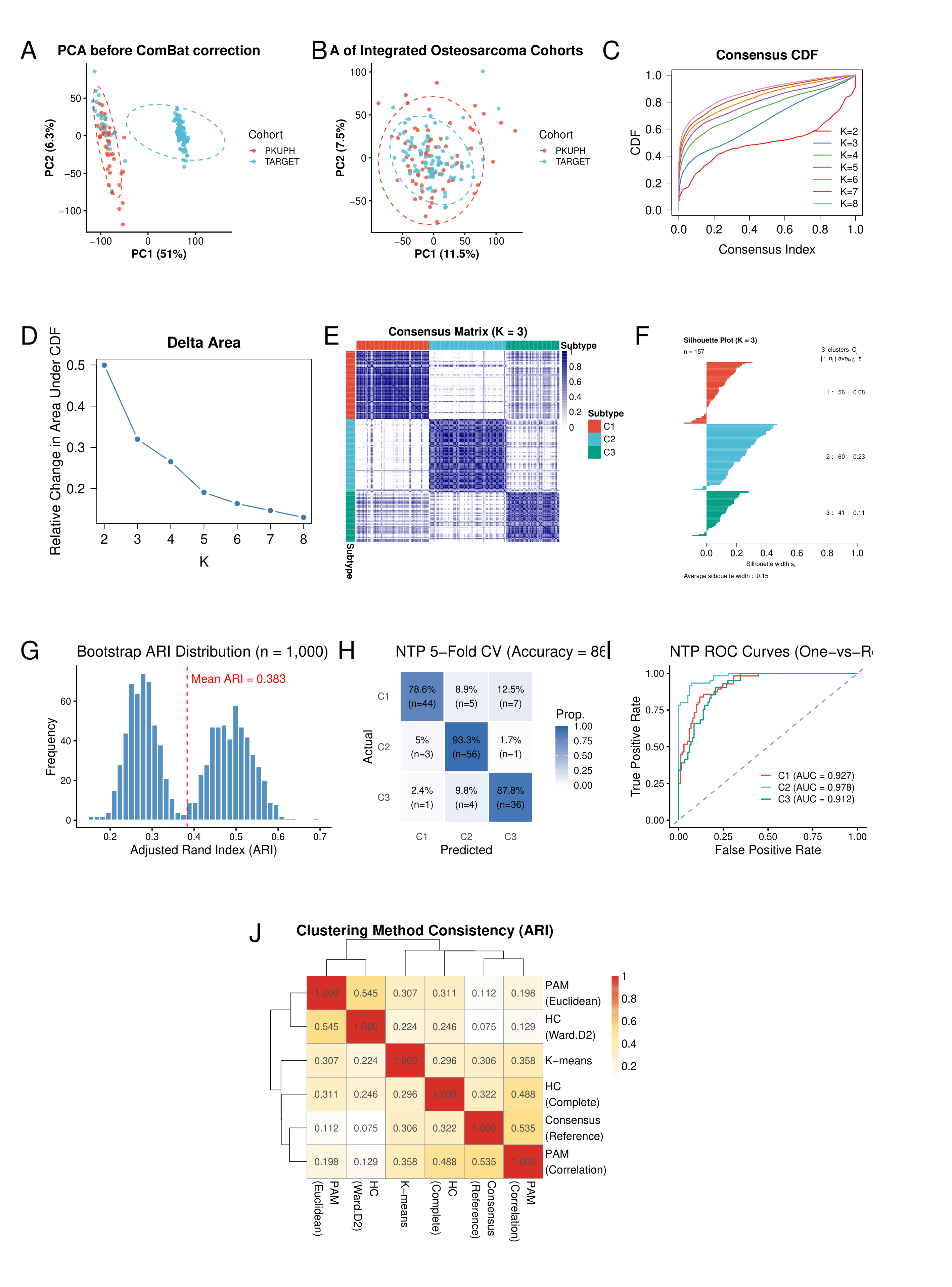

Supplement: Supplementary file 1 [file image1.tiff]

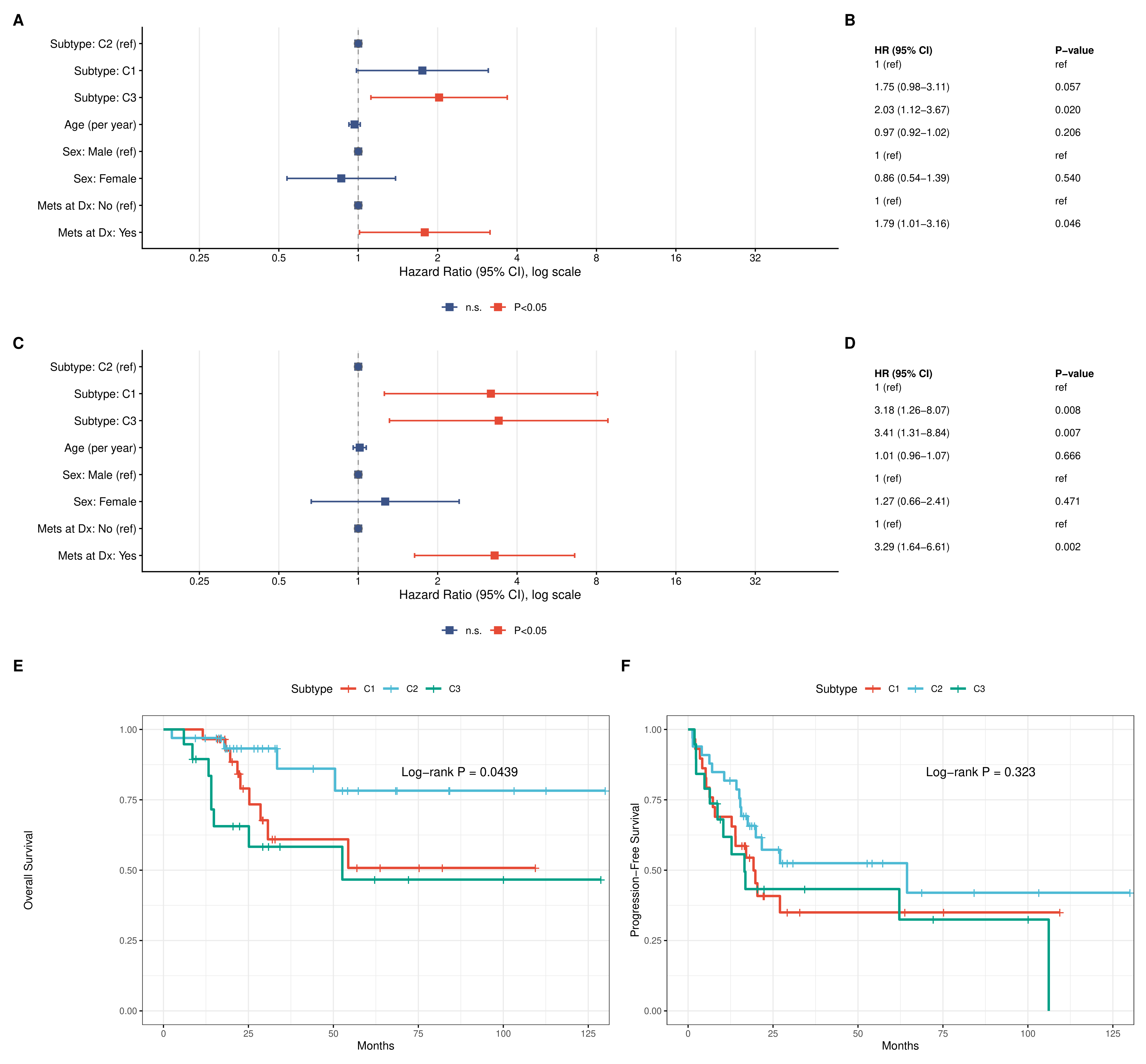

Supplement: Supplementary file 2 [file image2.tiff]

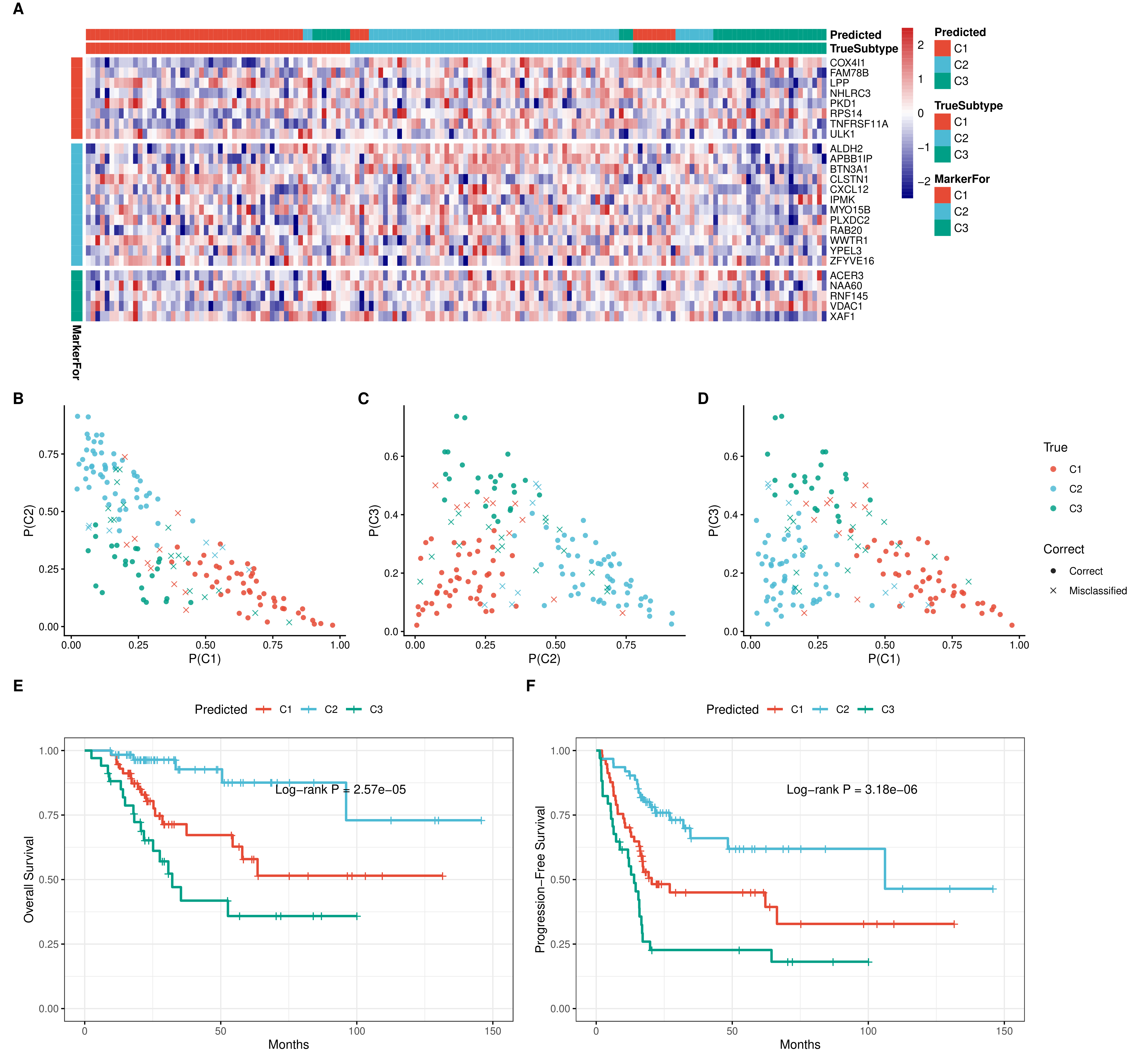

Supplement: Supplementary file 3 [file image3.tiff]

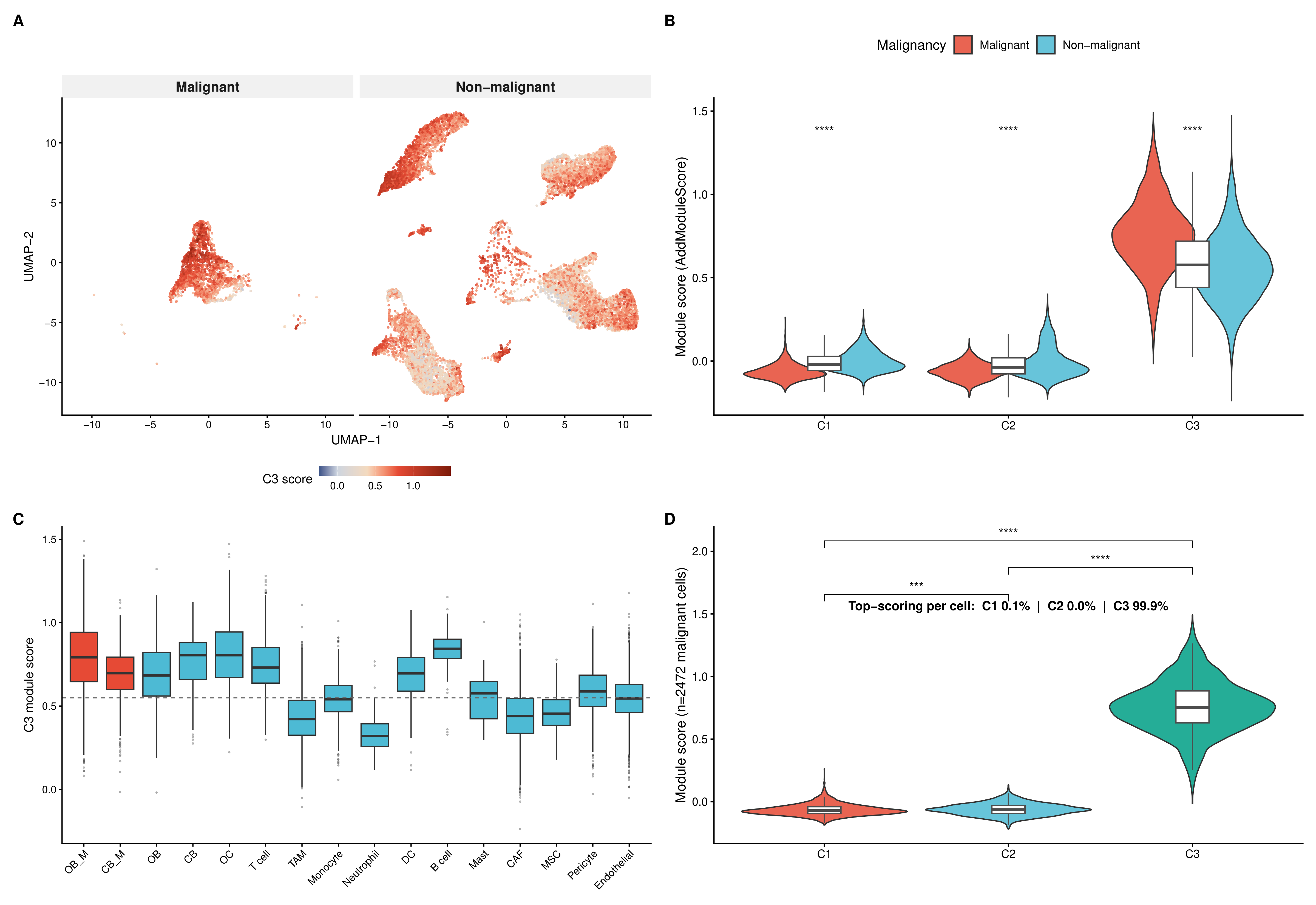

Supplement: Supplementary file 4 [file image4.tiff]
